# Speciation over the edge: gene flow among non-human primate species across a formidable biogeographic barrier

**DOI:** 10.1098/rsos.170351

**Published:** 2017-10-18

**Authors:** Ben J. Evans, Anthony J. Tosi, Kai Zeng, Jonathan Dushoff, André Corvelo, Don J. Melnick

**Affiliations:** 1 Biology Department, Life Sciences Building Room 328, McMaster University, 1280 Main Street West, Hamilton, ON, Canada L8S4K1; 2 Anthropology Department, Kent State University, 238 Lowry Hall, Kent, OH 44242, USA; 3 Department of Animal and Plant Sciences, University of Sheffield, Sheffield, UK; 4 New York Genome Center, 101 Avenue of the Americas, New York, NY 10013, USA; 5 Department of Ecology, Evolution, and Environmental Biology, Columbia University, 10th floor Schermerhorn Extension, 119th Street and Amsterdam Avenue, New York, NY 10027, USA

**Keywords:** mechanisms of speciation, gene flow, X chromosome, genomics, primate evolution, Wallace’s Line

## Abstract

Many genera of terrestrial vertebrates diversified exclusively on one or the other side of Wallace’s Line, which lies between Borneo and Sulawesi islands in Southeast Asia, and demarcates one of the sharpest biogeographic transition zones in the world. Macaque monkeys are unusual among vertebrate genera in that they are distributed on both sides of Wallace‘s Line, raising the question of whether dispersal across this barrier was an evolutionary one-off or a more protracted exchange—and if the latter, what were the genomic consequences. To explore the nature of speciation over the edge of this biogeographic divide, we used genomic data to test for evidence of gene flow between macaque species across Wallace’s Line after macaques colonized Sulawesi. We recovered evidence of post-colonization gene flow, most prominently on the X chromosome. These results are consistent with the proposal that gene flow is a pervasive component of speciation—even when barriers to gene flow seem almost insurmountable.

## 1. Background

### 1.1. Wallace’s Line and the drivers of speciation

A species is a group of reproductively compatible individuals with ancestor–descendant relationships that evolve through time [1]. Early ideas about the drivers of speciation recognized geographical isolation as an important prezygotic barrier to reproduction that contributes to this process, with this reasoning being heavily influenced by zoogeographic patterns (e.g. [2,3]). One particularly influential pattern is the sharp faunal transition that occurs between the islands of Borneo and Sulawesi, across ‘Wallace’s Line’ [4,5]. Many vertebrate genera do not span this barrier [6] and it is generally thought that many species in this region evolved in allopatry [7–10] as a consequence of a dynamic history of connectivity or isolation of large landmasses [11]. However, our understanding of the drivers of speciation here and elsewhere is in flux, including recent reappraisals of the degree to which gene flow exists among closely related species (e.g. [12]), the evolutionary consequences of gene flow and adaptation during speciation (e.g. [13]) and the degree to which geographical isolation is associated with ‘extrinsic’ (abiotic) versus ‘intrinsic’ (ecological) barriers to reproduction [14–16]. Wallace’s Line is an important evolutionary arena for studying speciation because some groups have anomalous distributions that span this barrier. These groups permit us to test whether allopatric lineages on either side of a precipitous biogeographic barrier are in fact isolated genetically and, if not, what genomic regions were affected by gene flow, and what were the adaptive implications.

### 1.2. Macaque monkeys have an anomalous distribution across Wallace’s Line

Macaque monkeys (*Macaca*) have the largest distribution of all non-human primate genera [17] and are among the most diverse [18]. Although they originated in Africa [19], almost one-third of macaque species occur just east of Wallace’s Line on the island of Sulawesi, which at less than 200 000 km^2^ comprises less than 4% of the geographical distribution of macaques [17]. The Sulawesi macaques are endemic to this island, allopatrically distributed and differentiated in behaviour [20,21], cranial morphology [22], pelage and other aspects of external morphology [23], and genetic variation [24,25]. Molecular studies support monophyly of the Sulawesi macaques and a sister relationship to the pigtail macaque, although phylogenetic relationships among the Sulawesi macaques remain poorly resolved (e.g. [25–28]). That macaques dispersed at least once across Wallace’s Line opens the possibility that it happened multiple times, and that variation among genomic regions could exist in the levels of gene flow across this biogeographic divide. To explore this possibility, we used restriction site-associated DNA (RADseq) data to characterize phylogenetic relationships and molecular variation among macaques in this geographical region, and we then used whole genome-sequence (WGS) data from one individual from each of three species to test for evidence of gene flow across Wallace’s Line.

## 2. Material and methods

### 2.1. Molecular data

**Figure 1.**
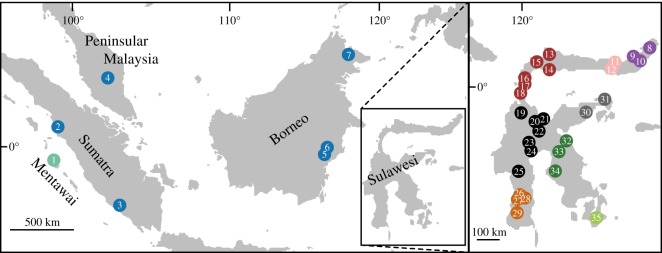
Thirty-five geographical origins of the 40 genetic samples analysed in this study. Numbered localities correspond to the approximate geographical origins of samples as follows, with asterisks denoting samples for which precise provenance is unknown: (1) *M. siberu*, (2) Ngasang, (3) Kedurang, (4) Malay*, (5) PM665, (6) PM664*, (7) Sukai, Gumgum, (8) PF660, (9) PF1003, (10) PF1001*, (11) PM1000, (12) PF654, (13) PF648, (14) PF651, (15) PM645, (16) PM639, (17) PF644, (18) PF643, (19) PF515, (20) PM565, PM566, PM567, (21) PM561, (22) PM582, (23) PM584, (24) PM592, (25) PM602, (26) PM613, (27) PM618, (28) PM614, PF615, PM616, (29) PF713, (30) PF549, (31) PM545, (32) PM571, (33) PM596, (34) PF625, (35) PF707. Dots are coloured by species as detailed in figures 2 and 3.

To perform tests for gene flow discussed below, it was necessary to establish a phylogenetic framework for our samples. We therefore generated and analysed RADseq [29] data from genetic samples of 40 individuals from 10 macaque species including all Sulawesi macaques and *Macaca nemestrina* and *M. siberu* from several sites in the Sunda Region (Borneo, Peninsular Malaysia, Sumatra and the Mentawai Islands). Genetic samples used in this study were collected from captive individuals as previously described [25,30] and the geographical origins of these samples are depicted in figure 1. Additional details on these samples and on macaque taxonomy are presented in the electronic supplementary material.

Two RADseq libraries were prepared by Floragenex (http://www.floragenex.com/). The first library was previously reported and constructed from nine *M. tonkeana* samples [31], and was sequenced using one Illumina HiSeq 2500 lane and 100 base pair (bp) paired-end reads. The second library has not previously been reported, included 31 new samples and was sequenced on one Illumina HiSeq 2500 lane with single-end 100 bp reads. Because reverse reads were available for only nine individuals, we restricted our analysis to the forward reads from these RADseq data.

In addition to RADseq, WGS was performed on three of these samples with a specific aim of testing for evidence of gene flow across Wallace’s Line. WGS data were collected for one male *M. nemestrina* (PM664), one male *M. tonkeana* (PM592) and one female *M. nigra* (PF660) using the Illumina HiSeqX platform with paired-end 150 bp reads.

### 2.2. Read filtering and quality control

RADseq data were de-multiplexed, trimmed and filtered using the process_radtags program of stacks v. 1.21 [32,33]. Reads were initially truncated to 75 bp; miscalled barcodes that differed by up to three mutations from only one barcode were rescued, and those with uncalled bases or bases with an average Phred-scaled quality score lower than 10 were removed. All reads were then filtered again with trimmomatic v. 0.36, removing overrepresented sequences that were identified using fastqc [34] and requiring retained sequences to have a minimum length of 36 bp and an average Phred-scaled quality score of at least 15 in a sliding window of 4 bp. After filtering repetitive regions (described below), the number of mapped RADseq reads per individual ranged from a low of 256 400 (for *M. maura* individual PM613) to a high of 6 346 894 (for *M. tonkeana* individual PM592) with an average and standard error of 2 016 557 and 301 929 reads per individual, respectively.

### 2.3. Genotyping and data filtering

For both RADseq and WGS data, the ‘MEM’ algorithm of bwa v. 0.7.8 [35] was used to map reads from each individual to a rhesus reference genome (rhemac2) which was downloaded from the University of California Santa Cruz Genome Browser (https://genome.ucsc.edu/). For the RADseq data, coverage of mapped reads ranged from a minimum of 7X (for *M. nigrescens* individual PF654) to a maximum of 83X (for *M. tonkeana* individual PM603), with an average and standard error of 36X and 4X, respectively. For the WGS data, coverage was greater than 40X for each of the three samples.

The Genome Analysis Toolkit (gatk) v. 3.6 [36] was used to perform genotyping and filtering as recommended by the ‘Best Practices’ pipeline [37,38]. Example command lines for initial generation of the WGS genotypes are provided in the electronic supplementary material. This included realignment of insertion/deletion (indel) polymorphisms with the RealignerTargetCreator and IndelRealigner functions. Genotyping was performed with the HaplotypeCaller and GenotypeGVCFs functions using, respectively, the EMIT_ALL_CONFIDENT_SITES and includeNonVariantSites functions of these commands. After this, the VariantFiltration and SelectVariants functions of gatk and a perl script were used to identify and remove positions that spanned an indel plus a buffer of 3 bp in both directions, repetitive regions identified in the reference genome by RepeatMasker [39], and individual genotypes that had coverage of less than 5X.

The WGS data were handled somewhat differently from the RADseq data in order to accommodate the different nature of these data (paired-end rather than single-end, shotgun sequencing rather than RADseq). Instead of trimming the WGS data with trimmomatic, we relied on the bwa MEM algorithm to softclip adapter sequences and used the BaseRecalibration function of gatk to recalibrate quality scores, excluding from the error model variant positions that were pre-called using Haplotypecaller. We did not perform base recalibration on the RADseq data because we performed stricter quality filtering on those data than the WGS data prior to mapping. Additionally, we did not perform de-duplication on the RADseq data because most of these data were single-end reads; for the WGS data de-duplication was performed with the MarkDuplicates function of picard (http://broadinstitute.github.io/picard).

Because our RADseq and WGS data included a mixture of male and female individuals, a haploid genotype was inferred for all sites on the X chromosome based on the allele with the highest depth of coverage (hereafter we refer to this as ‘X chromosome genotyping by depth of coverage’). For heterozygous sites, the single nucleotide polymorphism (SNP) with the highest coverage was used; if two SNPs had equal coverage, one was randomly selected. We also performed other approaches to filter and genotype the X chromosomes of males and females, which are discussed in the Results and discussion section, with similar results. For all analyses, we assumed that sites that mapped to the rhesus X chromosome also are on the X chromosome of the other macaque species we examined, and the same for the autosomes.

### 2.4. Phylogenetic and principal components analysis of restriction site-associated DNA data

Phylogenetic analysis of the RADseq data was performed using iqtree v. 1.5.0a [40] on the concatenated RADseq data from the autosomes and also a separate analysis of the RADseq X chromosome data. For both datasets, outgroup sequences were included from a human and an anubis baboon (genome assemblies hg19 and papAnu2, with the genome alignment to the rhesus macaque obtained from the University of California Santa Cruz Genome Browser or generated using lastz [41], respectively, as described in Evans et al. [31]). For the analysis of autosomal DNA, iqtree selected the general time reversible model with *Γ* distributed rate heterogeneity based on the Bayesian information criterion (BIC). For the analysis of X chromosome DNA, iqtree selected the TVM model of evolution with a proportion of invariant sites and a *Γ* distributed rate heterogeneity. For both analyses, node support was evaluated using the ultrafast bootstrap approach as implemented by iqtree. Because this analysis involves concatenated data, divergence times may not appropriately accommodate incomplete lineage sorting (ILS), and this aspect of the analysis of autosomal data is intended for qualitative rather than quantitative purposes. The phylogenetic analysis of the X (featured in the electronic supplementary material) carries the additional caveat that it was performed on a subset of the intra-individual molecular polymorphism in each female, using only the variant at each polymorphic site with the highest depth of coverage, as described above.

The program mcmctree, which is part of the paml v. 4.8 software suite [42], was then used to convert the maximum-likelihood (ML) topologies obtained from iqtree to a chronogram. The independent evolutionary rates model was used, and the analysis included only those data that had no heterozygous sites or missing data. For the autosomal and X chromosome analyses, the HKY85+*Γ* model of nucleotide substitution was deployed—of the models implemented by mcmctree, this was the most similar to the model selected by the BIC in iqtree. The alpha parameter for the *Γ* distribution was set to the ML estimate of 0.2176 which was recovered from iqtree for the autosomes and 1.106 for the X. For calibration of both analyses, the 95% confidence interval (95% CI) for the age of the Old World monkeys and apes (Catarrhini) was set to a lower and upper bound of 28 and 36 million years ago (Ma) and the divergence time of baboons and macaques was set to a lower and upper bound of 10 and 13 million years ago (Ma), following Finstermeier et al. [43]. To expedite the mcmctree analysis of the autosomal DNA only, the ‘cleandata’ option was used to exclude sites with ambiguous or missing data.

To further visualize genetic relationships among these data, we performed a principal components analysis (PCA) on the filtered autosomal RADseq genotypes using the program snprelate [44]. The SNPs were re-coded based on the dosage of the reference allele for all variant sites (‘method =copy.num.of.ref’) and were pruned to include only SNPs from sites with no missing data and that had a linkage disequilibrium threshold of 0.2 or less based on the composite measure of linkage disequilibrium [45] within a genomic window of size 500 000 bp. We performed a PCA on the full RADseq autosomal dataset and also on a reduced RADseq autosomal dataset including only the Sulawesi macaques. We also performed analyses of polymorphism on the X and autosomes as described and presented in the electronic supplementary material.

### 2.5. Gene flow analysis of whole genome-sequence data and divergence

If no gene flow occurred after macaques colonized Sulawesi from Borneo, phylogenetic analyses presented below indicate that most genomic regions of the Sulawesi macaques would be expected to be monophyletic with respect to the pigtail macaque *M. nemestrina*, with the exception of regions with ILS. ILS is expected to cause some genomic regions in a Sulawesi macaque to be more closely related to *M. nemestrina* than to other Sulawesi macaques (i.e. paraphyly of genetic variation in Sulawesi macaques), even though Sulawesi macaques are monophyletic over most of their genome. If gene flow occurred among macaques on either side of Wallace’s Line after macaques reached Sulawesi, there might be an excess of derived mutations (based on comparison to an outgroup genome) that are shared by a pigtail macaque from eastern Borneo and a macaque from western Sulawesi (e.g. *M. tonkeana*) when compared with derived sites that are shared between a pigtail macaque and a macaque from eastern Sulawesi (e.g. *M. nigra*). This expectation forms the basis of the D-statistic, which is also known as the ABBA-BABA test [46–50].

We hypothesized that if gene flow did occur between macaques on either side of Wallace’s line, then it would more likely be between the pigtail macaque (*M. nemestrina*) on Borneo and the tonkean macaque (*M. tonkeana*) on west central Sulawesi than between the pigtail macaque and the Celebes crested macaque (*M. nigra*) from the northeast tip of Sulawesi, because the first species pair are geographically closer to each other than the second species pair. To test this, we calculated the D-statistic and also a modification of the admixture fraction *f* proposed by Green et al. [46], called *f*_*DM*_, which was calculated as described on page 8 of the electronic supplementary material of Malinsky et al. [51]. *f*_DM_ is distributed on the interval [−1,1] and, under the null hypothesis of no introgression after colonization of Sulawesi, this statistic should be symmetrically distributed around zero. If the relative rate of gene flow is higher between *M. nemestrina* and *M. tonkeana* than between *M. nemestrina* and *M. nigra*, then *f*_DM_ will be greater than zero. Alternatively, if the opposite is true, *f*_DM_ will be less than zero. The null hypothesis that *f*_DM_ is equal to zero was evaluated using the weighted block jackknife approach [46] with *f*_DM_ values in non-overlapping 5 000 000 bp genomic regions that were weighted by the sum of the numbers of ABBA and BABA sites ([46] and defined below) in each window. For the autosomes, the gene flow statistics were calculated from heterozygous and homozygous genotypes. For the X chromosome, these statistics were calculated from variants from each individual with the highest depth of coverage as described above. We also explored other genotyping approaches for the X chromosome WGS data that are detailed below. Justification for genotyping the X chromosome by depth, at least in males, was based in part on the identification of pseudoheterozygous genotypes in the non-pseudoautosomal region of the X when diploid genotypes were inferred for this region. To illustrate the number of positions with shared and unshared heterozygous genotypes across individuals in the non-pseudoautosomal region of the X, a Euler diagram was generated using the R package ‘eulerr’ [52]. For all of these analyses, sites with missing genotypes were excluded.

Divergence was calculated as the average per-site per cent nucleotide difference between both alleles carried by two individuals. Except where stated in the electronic supplementary material, tables, divergence is presented without correction for multiple substitutions.

### 2.6. Testing for contamination

As a rough assessment of potential sample contamination by human DNA, which could potentially affect our inferences of gene flow, 1% of the WGS read data from each sample were randomly selected and classified against an index containing 12 full primate genomes (*Microcebus murinus*, *Chlorocebus sabaeus*, *Nomascus leucogenys*, *Callithrix jacchus*, *M. fascicularis*, *M. mulatta*, *Papio anubis*, *Gorilla gorilla gorilla*, *Pan paniscus*, *P. troglodytes*, *Pongo abelii*, *Homo sapiens*), using taxMaps [53]. With an aim of minimizing false positives due to (i) sequence similarity between macaques and these other species and (ii) the more complete sequencing coverage of the human reference genome, we used a strict paired-end classification mode that requires both mates to be mapped, and opted to compute the ‘lowest common ancestor’ between the independent classification of each mate in the pair.

## 3. Results and discussion

### 3.1. Evolutionary patterns among Southeast Asian macaque monkeys

**Figure 2.**
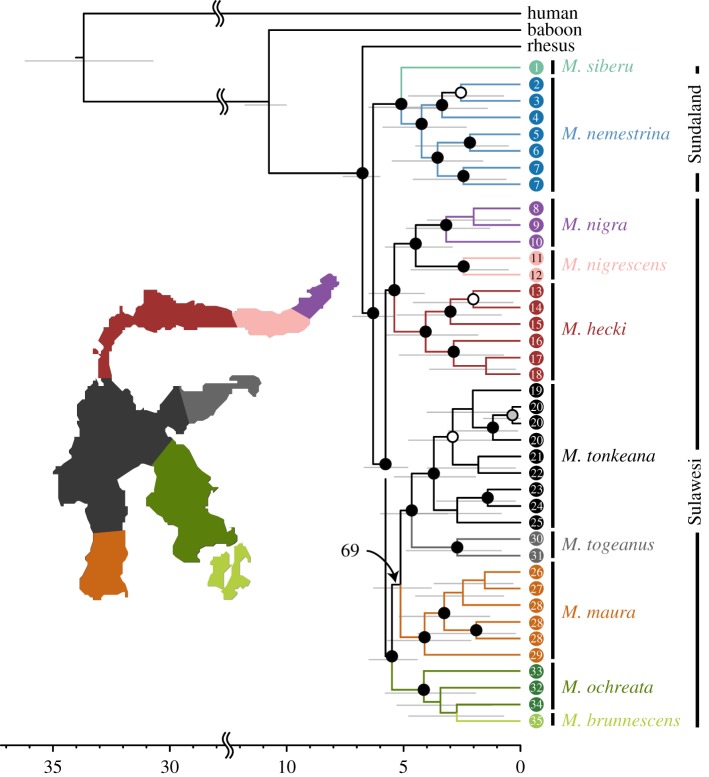
Time-calibrated phylogeny (chronogram) recovered from analysis of autosomal RADseq data. Scale indicates divergence in million years ago (Ma). Black, grey, and white dots over nodes reflect ultrafast bootstrap values from iqtree that are greater than 99, 95 and 90, respectively. Grey bars near each node indicate the 95% CI for divergence times recovered from mcmctree. An inset indicates ranges of Sulawesi macaques and low bootstrap support for one node is indicated with an arrow. Tips are numbered according to their geographical localities depicted in figure 1.

Despite a large amount of missing data (electronic supplementary material), the ML topology recovered from autosomal RADseq provided strong statistical support for relationships among Southeast Asian macaques that clustered by species and geographical region, and with unprecedented statistical support for relationships among Sulawesi macaques (figure 2). Well-supported relationships include strong support for monophyly of *M. nemestrina* + *M. siberu*, and for monophyly of the Sulawesi macaques. Within the Sulawesi macaques, two clades correspond, respectively, to species in the north peninsula and species in the rest of Sulawesi. Within the strongly supported clade that includes macaques from the rest of Sulawesi, there is poor resolution among three well-supported clades which include (i) *M. maura*, which occupies the southwestern peninsula, (ii) (*M. ochreata* + *M. brunnescens*), which occupy the southeast peninsula and surrounding islands, and (iii) (*M. tonkeana* + *M. togeanus*), which occur in central/central eastern Sulawesi.

Geographical structure of phylogenetic relationships is observed within species as well. For example, samples from the southern and eastern portions of the range of *M. hecki* each form a clade, and samples from southern and northern portions of the range of *M. tonkeana* also each form a clade. The only species that was not monophyletic in this analysis is *M. ochreata*, the southernmost sample from which forms a weakly supported clade with the *M. brunnescens* sample. Divergence estimates point to a similar timing of divergence of extant Sulawesi macaques from each other, and of *M. siberu* from *M. nemestrina*, both of which occurred approximately 5–6 Ma (figure 2). This corresponds with the earliest fossil evidence of Asian macaques approximately 5.5 Ma [54].

Phylogenetic analysis of the X chromosome RADseq data also recovered strong phylogenetic support for monophyly of the Sulawesi macaques with respect to *M. nemestrina* and for monophyly of the Sulawesi macaques of the northern peninsula with respect to the rest of Sulawesi (electronic supplementary material, figure S1). Some differences in phylogenetic relationships were inferred in this analysis compared to the autosomes, but none were well supported. Polymorphism on the X chromosome was lower than expected in four species with population sampling, but did not depart from expectations after allowing for a dynamic demography and selection on GC content using an ML model (model described in electronic supplementary material, tables S3–S11 and figure S2, and in [31]).

**Figure 3.**
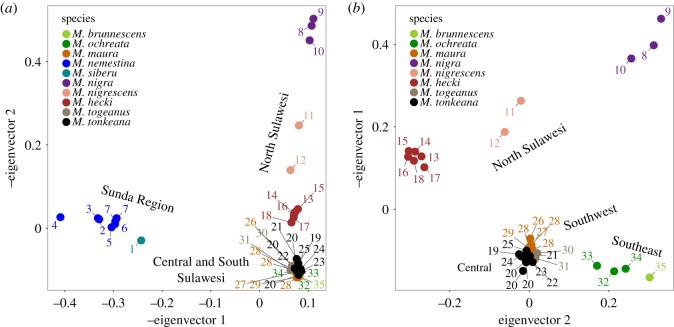
PCA analysis for all RADseq data (*a*) and RADseq data from only Sulawesi (*b*). Individual samples are numbered according to their geographical origins depicted in figure 1.

Similarly to the phylogenetic analysis, PCA of the autosomal RADseq data clustered samples by species and geographical origin (figure 3). The first PCA considered 2845 variable positions that had no missing data and low or no linkage disequilibrium among the 40 samples. The first eigenvector, which accounted for 7.45% of the variation in the data, separated variation in macaques from the Sunda Region and Sulawesi. The second eigenvector, which accounted for 4.85% of the variation in the data, separated variation in individuals of the northern peninsula of Sulawesi from the remainder of Sulawesi.

When autosomal RADseq data from only Sulawesi samples were analysed by a second PCA, 2969 variable positions had no missing data and low or non-existent level of linkage disequilibrium among the 32 samples (figure 3). The first eigenvector accounted for 6.88% and separated macaques of the northern peninsula from the remainder of Sulawesi, and also within the northern peninsula. The second eigenvector accounted for 5.86% of the variation and further separated geographical variation in macaques within each of these regions of Sulawesi.

Overall, these analyses provide strong support for monophyly of the Sulawesi macaques across the autosomes and X chromosomes, a strong correspondence between geography and macaque phylogenetic relationships on Sulawesi, and identify a non-significant dearth of molecular variation on the X chromosome.

### 3.2. Gene flow across Wallace’s Line, especially on the X

Using WGS data from three individuals and a rhesus macaque genome sequence as an outgroup, a significant excess of shared derived sites was observed between *M. nemestrina* from Borneo and *M. tonkeana* compared to between *M. nemestrina* and *M. nigra* on the X chromosome, but not on the autosomes. This excess of shared derived sites spans Wallace’s Line, even though most variation throughout the genome strongly supports monophyly of the Sulawesi macaques in the autosomes and the X (electronic supplementary material, figure 2).

On the X, the *f*_DM_ statistic was positive, which is consistent with gene flow between *M. nemestrina* and *M. tonkeana*; the magnitude and confidence intervals varied depending on the filters applied, and some combinations of filters and outgroups did not recover *f*_DM_ statistics that departed significantly from zero. However, for reasons discussed below, we view some of these analyses to be conservative. After removing repetitive regions and genotyping the X chromosome of males and females based on depth, the *f*_DM_ statistic was 0.3049 (95% CI: 0.1675–0.4423). Because these values have only one nucleotide called per site per individual, the values of Patterson’s *D* are the same as *f*_DM_. In these X chromosome data, there were 40 304 BBAA sites, 3781 ABBA sites and 2014 BABA sites, which, respectively, refer to sites with shared derived nucleotides in *M. tonkeana* and *M. nigra* (‘BBAA’ sites), *M. tonkeana* and *M. nemestrina* (‘ABBA’ sites), or *M. nigra* and *M. nemestrina* (‘BABA’ sites). When autosomal sites were considered, there was also an excess of shared derived sites between *M. nemestrina* from Borneo and *M. tonkeana*, but this was not significantly greater than zero. The autosomal *f*_DM_ statistic was 0.0042 (95% CI: −0.0065–0.0148) and Patterson’s *D* was 0.0027 (95% CI: −0.0044–0.0098). In the autosomal data, there were 1 203 957 BBAA sites, 145 277 ABBA sites and 144 491 BABA sites.

One concern in this analysis is that the *M. nigra* individual was a female, whereas the *M. nemestrina* and *M. tonkeana* individuals were male, raising the possibility that there was some sort of systematic bias in genotyping the X chromosome of male and female individuals. For this reason, we explored the effect of deleting sites for which a heterozygous genotype was recovered in the diploid genotype of males prior to genotyping sites on chromosome X based on the highest depth of coverage. After excluding these sites plus a 3 bp buffer, the *f*_DM_ statistic was lower but still significantly greater than zero: 0.0752 (95% CI: 0.0210–0.1293). This analysis had 39 825, 1938 and 1667 BBAA, ABBA and BABA sites, respectively. Significantly higher *f*_DM_ statistics were also recovered when diploid rather than haploid genotypes were used for the female in each of these analyses (electronic supplementary material, table S2). When baboons were used as an outgroup instead of the rhesus macaque and/or when the coverage cut-off was increased from 5X to 12X, a significantly higher *f*_DM_ statistic was recovered when all sites were considered, but not after excluding sites in which males had heterozygous diploid genotypes (electronic supplementary material, table S2). When we additionally excluded sites with heterozygous diploid genotypes in the female, a conservative measure based on the analysis of heterozygous genotypes on the X chromosome discussed below, the *f*_DM_ statistic was still positive in most of the analyses, although the 95% CIs overlapped zero (electronic supplementary material, table S2).

**Figure 4.**
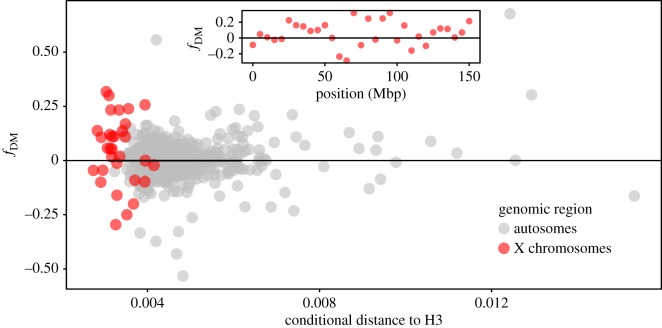
Conditional genetic distance to the *M. nemestrina* genome (H3) as a function of *f*_DM_ statistic for autosomes (grey, calculated including sites with heterozygous and homozygous genotypes) and the X chromosome (red, based on genotyping by depth after excluding positions with heterozyous diploid genotypes in males). Statistics are based on WGS data divided into non-overlapping windows of the reference genome spanning five million base pairs. Positive values of *f*_DM_ indicate an excess of derived sites (relative to the rhesus macaque) that are shared between the *M. tonkeana* (H2) genome and H3; negative values indicate an excess of derived sites that are shared between the *M. nigra* (H1) genome and H3. Genetic distances are ‘conditional’ in the sense that the uncorrected per cent of divergent sites between H2 and H3 or between H1 and H3 is plotted depending on whether *f*_DM_ is positive or negative, respectively, for each genomic window. Inset depicts *f*_DM_ in 5 Mbp windows on the X chromosome.

In figure 4, we present the results from chromosome X that were recovered after genotyping all individuals by depth after excluding positions with heterozyous diploid genotypes in males and using the rhesus macaque as an outgroup. The rhesus macaque sequence is generally a better outgroup for this analysis than the baboon sequence because it is more closely related to the ingroup taxa and therefore has fewer lineage-specific mutations. Additionally, the genome sequence is more complete for the rhesus macaque, so more data are considered when this species is set as an outgroup.

To further explore possible sex-specific genotype biases, we used information from [55] to infer that the boundary of the rhesus macaque pseudoautosomal region is at approximately position 403 495 of the chromosome X sequence in the rhemac2 rhesus macaque genome assembly. Of the pseudodiploid genotypes that were inferred from the male X outside of the pseudoautosomal region, a very small proportion of sites (0.046% or 0.048% for *M. tonkeana* and *M. nemestrina*, respectively) of the genotypes were heterozygous, and most of these sites were heterozygous in both males (electronic supplementary material, figure S3). In this same region of the X, twice as many genotypes (0.096%) of the female *M. nigra* were heterozygous, and most of these sites were not heterozygous in either male (electronic supplementary material, figure S3). These results are consistent with the proposal that many of the pseudoheterozygous genotypes in males arose as a consequence of mismapped reads from the Y chromosome, or other forms of genotyping error. By contrast, most of the heterozygous sites in the female appear to be real, although some of these also appear to be due to genotyping error (e.g. heterozygous positions on the non-pseudoautosomal region of the female chromosome X that are also heterozygous in both males). A summary of heterozygous non-pseudoautosomal region sites that had heterozygous genotypes in each individual is presented in electronic supplementary material, figure S3. Overall then, this analysis argues that most of the heterozygous genotype inferences in the non-pseudoautosomal region of the female X chromosome have a biological basis, as opposed to being genotype errors. Clearly, a useful direction for further study would be to analyse samples from only one sex in order to minimize, or at least homogenize, genotyping error across all samples in the analysis.

Another alternative explanation for a significant departure of ABBA-BABA statistics from zero is that there were different rates of evolution in each species on Sulawesi. For example, if *M. nigra* were to evolve more quickly than *M. tonkeana*, we would expect positions with a shared derived nucleotide in both Sulawesi species and also the pigtailed macaque (i.e. ‘BBBA’ sites) to evolve more frequently into CBBA sites than to BCBA sites, where ‘C’ refers to a derived site that evolved from ‘B’, which itself is a derived site compared to the rhesus outgroup sequence (which is designated ‘A’). To the extent that ‘C’ is a reversion to an ancestral ‘A’ nucleotide (which is expected in about one out of every three mutations), this could increase the number of apparent ABBA sites compared to BABA sites, and thus elevate the ABBA-BABA statistics (*f*_DM_, *D*) above zero without gene flow.

To explore this possibility, we quantified the number of CBBA and BCBA sites in the data with the expectation that they should be equivalent if there was no substantial difference in the rate of evolution between *M. nigra* and *M. tonkeana*. On the X, there were slightly more BCBA than CBBA sites (138 and 135, respectively), and there were 360 ‘CCBA’ sites (i.e. sites with three segregating nucleotides in which one had the highest frequency in both Sulawesi macaques, another had the highest frequency in the pigtailed macaque and a third was in the rhesus genome). This was also the case after removing sites in which males had heterozygous diploid genotypes (this analysis recovered 93 BCBA sites, 85 CBBA sites and 106 CCBA sites). On the autosomes, there also were slightly more BCBA than CBBA sites (5265 and 5212, respectively), and there were 7636 CCBA sites. These observations suggest that the rates of evolution in *M. tonkeana* and *M. nigra* were very similar; in fact, that there were slightly more BCBA than CBBA sites on the X and the autosomes make the ABBA-BABA statistics conservative with respect to the inference of gene flow between *M. tonkeana* and *M nemestrina*.

We also explored the possibility that variation in the level of contamination by human DNA could somehow influence the results of the ABBA-BABA statistics. Analysis with taxMaps suggested that the level of contamination of human DNA was very low (0.0061%, 0.0055% and 0.0065% of the reads from the *M. nemestrina*, *M. tonkeana* and *M. nigra* individuals, respectively). These proportions are not substantially different among the samples, and not substantially different in the male samples compared to the female. We suspect these percentages are overestimates caused by the more complete genome sequence of humans compared to the other species in this analysis.

The estimated divergence time between the Sulawesi macaques and *M. nemestrina* is 6 Ma (figure 2). Whether these genomic patterns are most consistent with a pulse of gene flow soon after Sulawesi was colonized by macaques, ongoing gene flow or some other scenario remains beyond the scope of this study. However, some general insights may be gleaned by examining divergence in putatively recently exchanged genomic regions. Recently, exchanged genomic regions are expected to have low divergence between the species that exchanged them, whereas anciently exchanged regions or regions with incomplete lineage sorting (ILS) are expected to have high divergence because more time has elapsed since the genomic region was shared. Interestingly, several genomic regions with high *f*_DM_ values also have relatively low divergence between *M. nemestrina* and *M. tonkeana* (figure 4). This again suggests against the relatively high *f*_DM_ values of these regions being due to ILS alone, and argues for further study of the nature and timing of possible gene flow across Wallace’s Line using WGS data (analyses of the RADseq data, not shown, provided insufficient statistical power for this analysis of gene flow). Taken together, these results open the possibility that gene flow occurred between macaques on either side of Wallace’s Line after the initial colonization of Sulawesi, and in a way that more profoundly affected variation on the X chromosome than the autosomes. A previous study did not recover evidence for gene flow across the Makassar Strait [26], although data analysed in that study (Sanger re-sequencing from a few dozen genic regions) would be unlikely to detect low levels of gene flow. Of note is that Evans *et al*. [26] detected paraphyletic molecular variation in the X-linked gene *TBL1X* in Sulawesi macaques, which could be a consequence either of ILS or gene flow.

Evidence of gene flow has been recovered from all major primate groups, including other papionins (e.g. [56]). Thus, the most striking aspects of this result are not that gene flow may have occurred between two primate species, but instead that (i) it may have occurred across such a precipitous biogeographic barrier, and (ii) its effect on the X chromosome may be more substantial than on the autosomes. There are several non-mutually exclusive scenarios that could explain this pattern. One possibility is that a low level of gene flow between *M. nemestrina* and *M. tonkeana* resulted in a small amount of shared variation in the autosomes and the X, and that this was followed by genetic drift or natural selection that increased the frequencies of some transferred regions on the X to a greater degree than on the autosomes. Indeed, strong effects of natural selection on the X have been reported in several other primates, including humans (e.g. [57–59]). A higher level of gene flow across the Makassar Strait on the X than the autosomes could also result if it was mediated mostly by female migration. But this would also be surprising because female papionin monkeys are generally philopatric [60] and this scenario is the opposite of expectations of a ‘large X effect’ in speciation [61], which predicts a lower level of gene flow between species on the X compared to the autosomes. Potentially relevant to these findings is the occurrence of interspecies hybridization between all parapatric species of Sulawesi macaque [24,62–66]. If gene flow across hybrid zones on Sulawesi were mediated mostly by male migration, then molecular varation introduced onto Sulawesi across Wallace’s Line would be expected to become more widely distributed (and thus less detectable by ABBA-BABA statistics) in the autosomes than on the X chromosome.

It is also possible that the significant departure of ABBA-BABA statistics from zero may in fact be due to factors other than gene flow. Although we did not find evidence that variation in the rate of evolution could account for this pattern, it is conceivable that population structure, for example due to isolation by distance, of the X chromosome existed in the ancestor of (*M. nemestrina* + the Sulawesi macaques) prior to dispersal of a subpopulation from Borneo to Sulawesi. If this were the case, the three whole-genome sequences we considered may not have captured a representative sample of molecular variation in each species, which could result in a misleading signal of gene flow. As stated above, it is also possible that natural selection or genetic drift could alter allele frequencies in such a way as to deliver a significant ABBA-BABA statistic. These possibilities could be explored with additional data from other individuals, and possibly allow genomic patterns that stem from ILS to be teased apart from those that stem from gene flow across Wallace’s Line.

## 4. Conclusion

In nature, speciation plays out in several dimensions: the geographical context ranges from allopatry to sympatry, gene flow varies from absent to extensive, and differentiation can be driven mostly by genetic drift or more prominently sculpted by natural selection. At first glance, speciation on either side Wallace’s Line appears to have unfolded largely with no gene flow across this barrier. However, our analyses of Southeast Asian macaque monkeys raise doubts about this assertion—at least for macaque monkeys—and provide several insights into diversification in this region, and to the process of speciation in general. Strong geographical structure of molecular variation in macaque RADseq data supports an important role of geography in regional faunal evolution. Thus, while Sulawesi Island may have been an archipelago in the past [11,67], the dispersal route of macaques among these palaeo-islands matches the modern geography of Sulawesi. We also identified a genomic signature of gene flow across Wallace’s Line, with the most pronounced signal on the X chromosome. This finding opens the possibility that gene flow can occur across formidable biogeographic barriers, and that in such cases it may vary in magnitude among genomic regions. Other explanations—such as demography, unsampled molecular variation and natural selection—are also plausible, and warrant further testing with additional samples. To the extent that gene flow across Wallace’s Line can be confirmed, this would contribute to examples of gene flow between species pairs in the genus *Macaca* (e.g. [24]), in other papionin genera (e.g. [56]) and in primate genera that are more closely related to humans (e.g. [68]).

## Supplementary Material

Supplementary Methods, Results, Tables, and Figures

## Data accessibility

All RADseq and WGS data and draft genome assemblies mapped to a rhesus macaque reference genome are deposited in the National Center for Biotechnology Information Short Read Archive accession numbers SRP041222, PRJNA398316 and PRJNA398316. Phylogenetic data, including trees and alignments, genotype files and scripts are deposited in Dryad as http://dx.doi.org/10.5061/dryad.3j218 [69]. Sampling locations are depicted in figure 1.
