# Correction to ‘Speciation over the edge: gene flow among non-human primate species across a formidable biogeographic barrier’

**DOI:** 10.1098/rsos.180736

**Published:** 2018-06-13

**Authors:** Ben J. Evans, Anthony J. Tosi, Kai Zeng, Jonathan Dushoff, André Corvelo, Don J. Melnick

*R. Soc. open sci.* **4**, 170351. (Published 18 October 2017). (doi:10.1098/rsos.170351)

The third accession number in the data accessibility section of the published paper is incorrect. The correct accession numbers are SRP041222, PRJNA398316 and PRJNA398198.

