# Supplementary material for: Speciation over the edge: gene flow among non-human primate species across a formidable biogeographic barrier
Source: R Soc Open Sci. 2017 Oct 18;4(10):170351. doi: 10.1098/rsos.170351 (PMC5666242; doi:10.1098/rsos.170351)
Supplement: Supplementary Methods, Results, Tables, and Figures [file rsos170351supp1.pdf]

Supplementary Information

Taxonomy of silenus group macaques

Macaques are divided into four species groups based on morphology [1, 2] and these groups each correspond to distinct phylogenetic clades [3]. The silenus group includes the liontail macaque *M. silenus*, which occurs in southwest India, and several species from Southeast Asia, which are the focus of this study, including the pigtail macaque (*M. nemestrina*) and the Sulawesi macaques: *M. tonkeana*, *M. maura*, *M. ochreata*, *M. brunnescens*, *M. hecki*, *M. nigrescens*, *M. nigra* [4, 5]. Within *M. tonkeana*, individuals in the west and east are significantly differentiated from each other [6] and the latter population is also known as *M. togeanus* [7]. *Macaca nemestrina*, as recognized by Fooden (1975), has been divided into several species including representatives from the Sunda Region (Sumatra, Borneo, Peninsular Malaysia – *M. nemestrina*), from the Mentawai Islands (*M. siberu* and *M. pagensis*; [8]), and from the northern portion of their range (*M. leonina*) [9]. Macaques, along with tarsiers and humans, are the only primates that have dispersed across Wallace's Line.

Genetic samples

RADseq data was collected from 40 samples in total, including representatives of all species from Sulawesi: *M. brunnescens* (1 female), *M. hecki* (4 females, 2 males), *M. maura* (2 females, 4 males), *M. nigra* (2 females, 1 male), *M. nigrescens* (1 female, 1 male), *M. ochreata* (1 female, 2 males), *M. tonkeana* (1 female, 8 males), and

M. togeanus (1 female, 1 male), seven *M. nemestrina* individuals, including four from Borneo (1 female, 3 males), two from Sumatra (both female), and one from Peninsular Malaysia (a female), and one female *M. siberu* from Siberut Island in the Mentawai Archipelago.

Data

The RADseq dataset had a substantial amount of missing data in an alignment of all samples; for autosomal DNA there was an average of 52.2% missing data per taxon (range 42.0% – 78.1%) and for the X there was an average of 42.8% (range 24.1% – 84.1%). Three individuals had over 70% missing data in the X and in the autosomal data: *M. maura* PM613, *M. nigrescens* PF654, and *M. ochreata* PM596. A summary of sequence data statistics for each sample is provided in Supplementary Table S1.

Genotyping

A general description of our bioinformatics pipeline is presented in the Methods. To supplement this, we include below examples of the commandlines used for generating the initial genotype files for the WGS data.

```
#ALIGN:
{bwa} mem -M -t 10 {reference} {R1_fastq} {R2_fastq} | {samtools} view
    -Shb -o {outputDir}/{lane}.mem.bam -

#SORTBAM:
```

```
{java} -Djava.io.tmpdir={javaTmpDir} -Xmx24576m -jar
    {picard}/AddOrReplaceReadGroups.jar MAX_RECORDS_IN_RAM=2000000
    CREATE_INDEX=true SORT_ORDER=coordinate VALIDATION_STRINGENCY=SILENT
    I={outputDir}/{lane}.mem.bam O={lane_bam} RGID={lane} RGLB={sample}
    RGSM={sample} RGPU="Unknown" RGCN={center} RGDS="RefVersion:rheMac2"
    RGPL="illumina"

#MERGE:
{java} -Djava.io.tmpdir={javaTmpDir} -Xmx24576m -jar
    {picard}/MergeSamFiles.jar USE_THREADING=true
    MAX_RECORDS_IN_RAM=2000000 CREATE_INDEX=true
    VALIDATION_STRINGENCY=SILENT INPUT={lane_bam_merge_string}
    OUTPUT={outputDir}/{sample}.merged.bam
{java} -Djava.io.tmpdir={javaTmpDir} -XX:ParallelGCThreads=5 -Xmx24576m
    -jar {picard}/MarkDuplicates.jar MAX_RECORDS_IN_RAM=2000000
    VALIDATION_STRINGENCY=SILENT CREATE_INDEX=true
    M={outputDir}/{sample}.dedup.metrics I={outputDir}/{sample}.merged.bam
    O={outputDir}/{sample}.dedup.bam

#REALIGN_CREATOR:
{java} -Djava.io.tmpdir={javaTmpDir} -Xmx24576m -jar {gatk} -T
    RealignerTargetCreator --interval_padding 200 -rf BadCigar -nt 4 -R
    {reference} -I {outputDir}/{sample}.dedup.bam -o
    {outputDir}/{sample}.forRealigner.intervals
```

```
#REALIGN:
{java} -Djava.io.tmpdir={javaTmpDir} -Xmx24576m -jar {gatk} -T
    IndelRealigner -dcov 1000 -rf BadCigar --consensusDeterminationModel
    USE_READS -R {reference} -targetIntervals
    {outputDir}/{sample}.forRealigner.intervals -I
    {outputDir}/{sample}.dedup.bam -o {outputDir}/{sample}.realigned.bam


#BQSR:
{java} -Djava.io.tmpdir={javaTmpDir} -Xmx24576m -jar {gatk} -T
    BaseRecalibrator --interval_padding 200 -rf BadCigar
    --downsample_to_fraction 0.1 -nct 4 -R {reference} -I
    {outputDir}/{sample}.realigned.bam -o {outputDir}/{sample}.recal.grp
    -knownSites {bqsr_sites_file}
{java} -Djava.io.tmpdir={javaTmpDir} -Xmx24576m -jar {gatk} -T PrintReads
    -nct 4 -rf BadCigar --disable_indel_quals --emit_original_quals -R
    {reference} -I {outputDir}/{sample}.realigned.bam -o
    {outputDir}/{sample}.final.bam -BQSR {outputDir}/{sample}.recal.grp


#HAPLOTYPECALLER:
{java} -XX:ParallelGCThreads=2 -Djava.io.tmpdir={javaTmpDir} -Xmx65536M
    -jar {gatk} -T HaplotypeCaller --genotyping_mode DISCOVERY -A
```

```
    AlleleBalanceBySample -A DepthPerAlleleBySample -A DepthPerSampleHC -A
    InbreedingCoeff -A MappingQualityZeroBySample -A StrandBiasBySample -A
    Coverage -A FisherStrand -A HaplotypeScore -A
    MappingQualityRankSumTest -A MappingQualityZero -A QualByDepth -A
    RMSMappingQuality -A ReadPosRankSumTest -A VariantType -A
    StrandOddsRatio --emitRefConfidence GVCF -l INFO -rf BadCigar -R
    {reference} -nct 1 -I {outputDir}/{sample}.final.bam -o
    {outputDir}/{sample}_{intervalName}.g.vcf -L {intervalValue}

#COMPRESS/INDEX:
{bgzip} -f {outputDir}/{sample}_{intervalName}.g.vcf
{tabix} -f -p vcf {outputDir}/{sample}_{intervalName}.g.vcf.gz

#MERGE/EMIT:
{java} -XX:ParallelGCThreads=2 -Djava.io.tmpdir={javaTmpDir} -Xmx65536M
    -jar {gatk} org.broadinstitute.gatk.tools.CatVariants -R {reference}
    --assumeSorted {vcf_list} -o {outputDir}/{sample}.g.vcf.gz
{java} -XX:ParallelGCThreads=2 -Djava.io.tmpdir={javaTmpDir} -Xmx65536M
    -jar {gatk} -T GenotypeGVCFs -R {reference} --variant
    {outputDir}/{sample}.g.vcf.gz -o {outputDir}/{sample}.vcf

#COMPRESS/INDEX:
{bgzip} -f {outputDir}/{sample}.vcf
{tabix} -f -p vcf {outputDir}/{sample}.vcf.gz
```

Alignments for phylogenetic analysis

The alignment of autosomal RADseq data, including gapped positions, was 10,639,115 bp (out of a total genome size of about 3 Gigabases), and 91.6% of these positions were invariant. For the X, the alignment including gapped positions was 217,762 bp, and 92.7% of these were invariant.

Divergence

Based on the WGS data, genome-wide divergence per site between either of the Sulawesi species and the pigtailed macaque was 0.5% for autosomal DNA and 0.3% for the X. Divergence between *M. tonkeana* and *M. nigra* was 0.3% for the autosomes and 0.2% on the X. Similar to the results from analysis of the X/A polymorphism ratio discussed below, a higher level of divergence on the autosomes compared to the X is consistent with faster male evolution.

The X/A polymorphism ratio

In a constant sized population with equal variance in reproductive success between the sexes, the null expectation for the relative level of polymorphism on the X and autosomes is 0.75 [10]. We used two approaches to test this null hypothesis, with our analyses focused on each of the four species or populations for which we had data from at least four individuals in the RADseq data. This included *M. tonkeana* (9 individuals), *M. maura* (5 or 6 individuals depending on the analysis; see below),

M. hecki (6 individuals), and *M. nemestrina* from Borneo (4 individuals). We excluded data from the east population of *M. tonkeana* (i.e., *M. togeanus*) and the *M. nemestrina* populations from Sumatra and Peninsular Malaysia to reduce the effects of population subdivision on the results.

As described in Evans et al. 2014, the first method standardized the ratio of pairwise nucleotide diversity per site (π) on the X over that of the autosomes using the Jukes-Cantor corrected (1969) divergence from baboons for the X and autosomes, respectively, and included a correction for ancestral polymorphism as detailed in Charlesworth and Charlesworth (2010). Because this method required no missing data within a species in order for a site to be considered, we excluded *M. maura* individual PM613 due to low coverage. The second method estimated the X/A polymorphism ratio (λ) using a model of evolution that included the possibility of a dynamic demographic history and natural selection on GC content (and/or GC-biased gene conversion), as described elsewhere [11, 14–17]. Thus, $N_{eX}=\lambda N_{eA}$, where N_{eX} and N_{eA} are the effective population sizes of the X and autosomes, respectively, and λ is not influenced by differences between these genomic regions in mutation rate. This model allowed for missing data, so no individuals were excluded within each of these species or populations. The models to which the data from each species were fitted have several parameters, and include a 'full' model in which all parameters are estimated independently, and several nested models in which one or more parameters are fixed to some constant, or set to be equivalent to one another. The full model included two time intervals with different effective population sizes with instantaneous change between the ancestral and recent population size occurring τ

generations ago, and the ratio of the current to ancestral population size being equal to ρ. To account for the possibility that natural selection acted on GC content, or the equivalent genomic effects of GC-biased gene conversion, we fitted the data to a model of evolution between two types of nucleotides: those with a weak bond (adenosines and thymines) and those with a strong bond (guanines and cytosines). The polymorphism data were recoded to include only variable sites in which a guanine (G) or a cytosine (C) nucleotide was segregating with an adenine (A) or a thymine (T). In these models, the parameters θ_{01} and θ_{10} refer to the mutation parameters from G or C nucleotides to A or T nucleotides, and the reverse, respectively, as detailed in Evans et al. 2014. The parameter γ reflects whether GC-biased gene conversion (gBGC) or natural selection on GC content favors an increase in GC content (a positive parameter value) or a decrease in GC content (a negative parameter value). In the full model, γ is estimated separately for the autosomes (γ_A) and the X (γ_X), and in some of the nested models γ_A and γ_X are set to be equivalent and/or equal to zero, which corresponds to no gBGC or neutrality of GC content. The polymorphism data were also fitted to an equilibrium model in which population size is constant and for which there is no X/A polymorphism ratio (λ) parameter. More detailed information and the statistical rationale for these models are available elsewhere [11, 14–17]. If the equilibrium model was poorly supported, weighted parameter estimates were then calculated across all models using AIC weights, as described by Wagenmakers and Farrell 2004.

Low molecular polymorphism on the X can be explained by demography and natural selection

Fig. S2 depicts the X/A polymorphism ratio calculated from standardized π using RADseq data from four species, after separating the polymorphism data into categories based on genomic position relative to annotated genes in the rhesus genome. Additional polymorphism statistics are presented in Supplementary Tables S3 – S6. As expected, diversity and divergence was similar in *M. tonkeana* to that previously reported based on an expanded dataset that included paired-end sequences [11], even though there were differences in the bioinformatic analyses of each study.

In the four species in which we assessed population genetic variation, *M. nemestrina* from Borneo was the most polymorphic. The 95% CIs for π and θ_{W} overlapped for the three Sulawesi species with population genetic data from at least 4 individuals (*M. tonkeana*, *M. maura*, and *M. hecki*). In genomic regions far from genes, which presumably are least affected by natural selection, the X/A polymorphism ratio was lower than expectations (Fig. S2). However, in these genomic regions Tajima's D of autosomal DNA was significantly negative (Tables S3 – S6), indicating an excess of low frequency polymorphisms. This could stem from population expansion, although in *M. maura*, the 95% CI for this parameter was near zero suggesting population size of that species may have varied less than that of the others.

That Tajima's D is significantly different from zero provides circumstantial evidence for a dynamic demography in at least some of these species, and changes in population size are known to influence the X/A polymorphism ratio [19]. Addition-

ally, other factors such as gBGC or natural selection on GC content have the potential to affect the X/A polymorphism ratio (e.g., Evans et al. 2014). For these reasons, we fitted the polymorphism data from genomic regions far (>51,000 bp) from genes to several models of evolution to polymorphism data from the X and autosome for each of the four populations or species for which we had data from >3 individuals. For the three species where the equilibrium model (with no change in population size over time) was not supported, the weighted average of parameter estimates over all models based on AIC weights are presented in Table S11. Parameter estimates for each model for each species or population are presented in Supplemental Tables S7 – S9.

M. maura was unusual among the 4 populations we tested because the equilibrium model was provided a relatively good fit to the data. This is illustrated by the AIC weight for the equilibrium model (∞) in Table S9 that is over twice as high as that of any other model. The other three populations/species each had evidence for a dynamic demography and zero weight for the equilibrium model.

For all species, gBGC and/or selection favoring increased GC content is supported because the the maximum likelihood estimates and/or model averages for the gBGC parameters on the autosomes and the X, γ_{A} and γ_{X} respectively, are greater than zero. This indicates that gBGC and/or selection for GC content favor an increase in GC content (Table S11). Moreover, all models that set γ_{A} or γ_{X} equal to zero had low AIC weights (Tables S7 – S9). If the strength of gBGC and/or selection favoring increased GC is similar on the X and autosomes, we expect $\gamma_{\mathrm{X}} = \lambda\gamma_{\mathrm{A}}$; for all four species/populations the AIC weights of these models were high compared

to the other models that relaxed this constraint, suggesting that the forces driving GC-biased molecular evolution in these genomic regions were indeed similar. This latter finding was also recovered previously for *M. tonkeana* [11] using an expanded dataset from that species that also analyzed paired end sequences from RADseq.

In this analysis, variable positions are re-coded into two states, A_0 and A_1, where A_0 refers to G or C nucleotides and A_1 refers to A or T nucleotides (see Methods). Additionally, $\theta_{01} = 4N_{\mathrm{e}}\mu_{01}$, where μ_{01} represents the mutation rate from $A_0 \rightarrow A_1$, and $\theta_{10} = 4N_{\mathrm{e}}\mu_{10}$, where μ_{10} represents the mutation rate from $A_1 \rightarrow A_0$. In all species, $\theta_{01} > \theta_{10}$ for the X and for the autosomes (Table S11). This suggests that in each of these different species there are more variable positions in which a G or a C is the major (more common) allele than where an A or a T is the major allele. Also of interest is the observation that in each species, $\theta_{\mathrm{ij}A}\ \lambda/\theta_{\mathrm{ijX}} > 1$, where ij is 01 or 10 and A and X refer to the autosomes and X respectively. This indicates that the mutation rate is higher in the autosomes than in the X, which is suggestive of male driven evolution – a result that is also suggested by pairwise divergence between the three species for which we performed WGS as described below.

Thus, while we recovered significantly lower polymorphism on the X than expected based on π in four macaque species, in each one this could be accounted for by an evolutionary model that includes a dynamic demography and selection on gBGC/natural selection on GC content. One factor not incorporated in these analyses and that is beyond the scope of this study is the possibility that hybridization among species via male dispersal could influence the X/A ratio. As discussed above, most papionin monkeys have female philopatry and hybridization has been detected

between all parapatric species pairs on Sulawesi. If hybridization were mostly mediated by male migration, diversity in the autosomes would be increased to a greater extent than the X. This possibility could explain the lower (but not significantly so) levels of diversity on the X. Added to this, other factors such as natural selection in males on deleterious recessive mutations, could also decrease diversity on the X.

References

[1] Fooden, J. Provisional classification and key to living species of macaques (Primates: *Macaca*). *Folia primatologica*, **25**, (1976) 225–236.

[2] Delson, E. Fossil macaques, phyletic relationships and a scenario of deployment. *The Macaques: Studies in ecology, behavior and evolution*, (1980) 10–30.

[3] Tosi, A. J., Morales, J. C., and Melnick, D. J. Paternal, maternal, and biparental molecular markers provide unique windows onto the evolutionary history of macaque monkeys. *Evolution*, **57**, (2003) 1419–1435.

[4] Fooden, J. Taxonomy and evolution of liontail and pigtail macaques (Primates : Cercopithecidae). *Fieldiana Zoology*, **67**, (1975) 1–169.

[5] Fooden, J. *Taxonomy and evolution of the monkeys of Celebes* Karger Basel, 1969.

[6] Evans, B., Supriatna, J., and Melnick, D. Hybridization and population genetics of two macaque species in Sulawesi, Indonesia. *Evolution*, **55**, (2001) 1686–1702.

[7] Froehlich, J. W. and Supriatna, J. Secondary intergradation between *Macaca maurus* and *M. tonkeana* in South Sulawesi, and the species status of *M. togeanus*. *Evolution and ecology of macaque societies*, (1996) 43–70.

[8] Roos, C., Ziegler, T., Hodges, J. K., Zischler, H., and Abegg, C. Molecular phylogeny of Mentawai macaques: taxonomic and biogeographic implications. *Molecular Phylogenetics and Evolution*, **29**, (2003) 139–150.

[9] Groves, C. P. *Primate taxonomy* Smithsonian Books, 2001.

[10] Charlesworth, B. Effective population size and patterns of molecular evolution and variation. *Nature Reviews Genetics*, **10**, (2009) 195–205.

[11] Evans, B. J., Zeng, K., Esselstyn, J. A., Charlesworth, B., and Melnick, D. J. Reduced representation genome sequencing suggests low diversity on the sex chromosomes of tonkean macaque monkeys. *Molecular Biology and Evolution*, **31**, (Sept. 2014) 2425–2440.

[12] Jukes, T. H. and Cantor, C. R. Evolution of protein molecules. *Mammalian Protein Metabolism*, **3**, (1969) 132.

[13] Charlesworth, B. and Charlesworth, D. *Elements of Evolutionary Genetics* Roberts Publishers, 2010.

[14] Haddrill, P. R., Zeng, K., and Charlesworth, B. Determinants of synonymous and nonsynonymous variability in three species of Drosophila. *Molecular Biology and Evolution*, **28**, (2011) 1731–1743.

[15] Zeng, K. and Charlesworth, B. Studying patterns of recent evolution at synonymous sites and intronic sites in Drosophila melanogaster. *Journal of Molecular Evolution*, **70**, (2010) 116–128.

[16] Zeng, K. and Charlesworth, B. The joint effects of background selection and genetic recombination on local gene genealogies. *Genetics*, **189**, (2011) 251–266.

[17] Zeng, K. and Charlesworth, B. Estimating selection intensity on synonymous codon usage in a nonequilibrium population. *Genetics*, **183**, (2009) 651–662.

[18] Wagenmakers, E.-J. and Farrell, S. AIC model selection using Akaike weights. *Psychonomic Bulletin & Review*, **11**, (2004) 192–196.

[19] Pool, J. E. and Nielsen, R. Population size changes reshape genomic patterns of diversity. *Evolution*, **61**, (2007) 3001–3006.

Supplementary Tables and Figures

Table S1: Information on sequence data analyzed in this study including the species (Species), sample identification number (SampleID), sex (Sex), number of reads before and after trimming (Untrimmed and Trimmed, respectively), read length after trimming if performed or before trimming for the HiSeqX data (Readlength), GC content (GC), and the number of mapped reads (mapped). For the HiSeqX data, trimming was not performed (np). Mapped reads were computed for RADseq and HiSeqX data by the flagstat command of SAMTOOLS and GATK, respectively.

Species	SampleID	Sex	Untrimmed	Trimmed	Readlength	GC	mapped
RADseq							
M. nemestrina	Gumgum	F	3609945	3503070	36-75	52	2413976
M. nemestrina	Kedurang	F	2811747	2686390	36-75	52	1837733
M. nemestrina	Malay	F	2888661	2820801	36-75	52	1926671
M. nemestrina	Ngasang	F	1863406	1819857	36-75	52	1250488
M. nemestrina	PM664	M	4794910	4720761	36-75	52	3242719
M. nemestrina	PM665	M	8785118	2447219	36-75	53	1666291
M. nemestrina	Sukai	M	2561836	2505653	36-75	53	1777480
M. siberu	pagensis	F	2374748	2315204	36-75	53	1602553
M. nigra	PF1001	F	1103174	1726287	36-75	53	1193708
M. nigra	PF660	F	1767769	1080813	36-75	53	744724
M. nigra	PM1003	M	2709319	2637075	36-75	53	1847731
M. nigrescens	PF654	F	3259548	911105	36-75	53	472852
M. nigrescens	PM1000	M	2860128	2778827	36-75	53	1935144
M. hecki	PF643	F	5770315	5628031	36-75	53	3888077
M. hecki	PF644	F	9630884	9306553	36-75	54	6627357
M. hecki	PF648	F	4852016	4768222	36-75	53	3322875
M. hecki	PF651	F	5038203	5035540	36-75	53	3509593
M. hecki	PM639	M	5269803	5154271	36-75	53	3551944
M. hecki	PM645	M	5855161	5746693	36-75	53	3974092
M. maura	PF615	F	5625181	5532103	36-75	53	3815996
M. maura	PF713	F	10698886	10557589	36-75	54	7524686
M. maura	PM613	M	935933	921611	36-75	53	637370
M. maura	PM614	M	4160191	4058140	36-75	53	2843009
M. maura	PM616	M	6667369	6526349	36-75	53	4530947
M. maura	PM618	M	4285281	4160860	36-75	54	2969422
M. tonkeana	PF515	F	11982906	11702493	36-75	53	8148982
M. tonkeana	PM561	M	11624883	11309233	36-75	53	7841954
M. tonkeana	PM565	M	12132625	11852221	36-75	53	8241475
M. tonkeana	PM566	M	9921261	9688908	36-75	53	6712122
M. tonkeana	PM567	M	11892242	11561026	36-75	53	8033420
M. tonkeana	PM582	M	12296341	11967241	36-75	54	8439642
M. tonkeana	PM584	M	12812865	12442854	36-75	53	8654276
M. tonkeana	PM592	M	13843609	13368052	36-75	54	9412709
M. tonkeana	PM602	M	13737591	13373987	36-75	54	9521922

Continued on next page

Continued from previous page

Species	SampleID	Sex	Untrimmed	Trimmed	Readlength	GC	mapped
M. togeanus	PF549	F	1096486	1070693	36-75	53	741166
M. togeanus	PM545	M	1783072	1762114	36-75	53	1239608
M. ochreata	PF625	F	2164485	2128202	36-75	53	1477886
M. ochreata	PM571	M	1687457	1667269	36-75	53	1169513
M. ochreata	PM596	M	2414864	2124502	36-75	54	685724
M. brunnescens	PF707	F	2277761	2217043	36-75	53	1567209
WGS							
M. nemestrina	PM664	M	949729200	np	151	41	927910917
M. nigra	PF660	F	916229358	np	151	41	894519506
M. tonkeana	PM592	M	913316498	np	151	41	892349062

Table S2: Gene flow statistics for the X chromosome calculated from data after different filtering steps, and using different outgroup taxa. The first panel indicates the f_{DM} statistic with confidence intervals in parentheses. The second panel indicates the number of ABBA, BABA, and BBAA sites in each analysis, with the definition of these site patterns provided in the main text. Each panel is divided into rows based on whether sites with heterozygous diploid genotypes were included and called as a haploid genotype based on depth (included), excluded if either or both males had a heterozygous diploid genotype (no male hets), or excluded if any individual had a heterozygous diploid genotype (no hets). For each of these filters, we calculated gene flow statistics after removing sites where any genotype had <5X or <12X coverage (5X or 12X respectively), using a rhesus or baboon outgroup (rhesus or baboon, respectively), and/or using a haploid genotype for all individuals or diploid genotype for the female all (all haploid or female diploid, respectively). In the top panel, asterisks indicate f_{DM} statistics whose confidence intervals are higher than zero, which is consistent with gene flow between *M. nemestrina* and *M. tonkeana*. In general, less data are considered by the analyses on the left and bottom of each panel.

	5X, rhesus, all haploid	5X, rhesus, female diploid	5X, baboon, all haploid	12X, rhesus, all haploid	12X, baboon, all haploid
f_{DM}					
included	0.30492 (0.16752 - 0.44232)*	0.38206 (0.28071 - 0.48342)*	0.16879 (0.07368 - 0.26390)*	0.31821 (0.17475 - 0.46167)*	0.16973 (0.07373 - 0.26573)*
no male hets	0.07517 (0.02101 - 0.12933)*	0.19022 (0.12465 - 0.25579)*	0.03392 (-0.01003 - 0.07788)	0.04351 (-0.00781 - 0.09483)	0.03408 (-0.00984 - 0.07801)
no hets	0.04690 (-0.01601 - 0.10980)	0.04726 (-0.01560 - 0.11012)	-0.01926 (-0.07222 - 0.03369)	0.04733 (-0.00489 - 0.09954)	0.03851 (-0.00474 - 0.08176)
ABBA;BABA;BBAA					
included	3781; 2014; 40304	4156.5; 2401.5; 40312	3123; 2221; 30999	3457; 1788; 36736	2898; 2057; 28566
no male hets	1938; 1667; 39825	2104.5; 1725; 39722.5	1905; 1780; 30349	1523; 1396; 35709	1608; 1502; 27426
no hets	1518; 1382; 38538	1518; 1381; 38538	1451; 1508; 29363	1527; 1389; 35705	1618; 1498; 27424

Table S3: Borneo *M. nemestrina* polymorphism ($n = 1$ female, 3 males). Data are divided into three categories based on their position relative to genes, including positions spanning genes ± 1000 bp in both directions (plusminus), positions 1000–51000 bp from genes (1000to51000), and positions >51000 bp from genes (51000plus). Statistics include the number of sites genotyped (Sites), the number of RAD tags (RADtags), the number of segregating sites (S), Watterson's θ (θ_W), pairwise nucleotide diversity (π), divergence from humans with Jukes-Cantor correction and correction for ancestral polymorphism (divergence), Tajima's D (TajD), and the number of singleton sites over the number of segregating sites (S_e/S). 95% confidence intervals are in parentheses.

Statistic	plusminus	1000to50000	51000plus
aDNA			
Sites	424889	756435	2471619
RADtags	4614	8242	27524
S	2862 (2759 - 2966)	5311 (5162 - 5463)	19342 (19048 - 19627)
θ_W	0.00260 (0.00250 - 0.00269)	0.00271 (0.00263 - 0.00279)	0.00302 (0.00297 - 0.00306)
π	0.00242 (0.00233 - 0.00251)	0.00254 (0.00246 - 0.00262)	0.00281 (0.00277 - 0.00285)
divergence	0.06065 (0.05989 - 0.06141)	0.06363 (0.06303 - 0.06421)	0.06637 (0.06601 - 0.06668)
π/divergence	0.040 (0.03829 - 0.04159)	0.040 (0.03869 - 0.04121)	0.042 (0.04165 - 0.04304)
TajD	-0.376 (-0.441 - -0.313)	-0.339 (-0.384 - -0.294)	-0.381 (-0.406 - -0.355)
Se/S	0.507 (0.489 - 0.526)	0.502 (0.488 - 0.516)	0.519 (0.512 - 0.526)
xDNA			
Sites	4803	10354	33621
RADtags	68	139	455
S	11 (5 - 18)	21 (13 - 31)	78 (61 - 96)
θ_W	0.00125 (0.00057 - 0.00204)	0.00111 (0.00068 - 0.00163)	0.00127 (0.00099 - 0.00156)
π	0.00118 (0.00056 - 0.00194)	0.00105 (0.00063 - 0.00153)	0.00124 (0.00097 - 0.00152)
divergence	0.05906 (0.05166 - 0.06691)	0.05166 (0.04712 - 0.05635)	0.06343 (0.06071 - 0.06622)
π/divergence	0.020 (0.00924 - 0.03310)	0.020 (0.01208 - 0.03062)	0.020 (0.01507 - 0.02428)
TajD	-0.558 (-0.847 - 0.083)	-0.557 (-0.857 - -0.117)	-0.216 (-0.489 - 0.082)
Se/S	0.909 (0.727 - 1.000)	0.905 (0.762 - 1.000)	0.795 (0.705 - 0.872)

Table S4: *M. tonkeana* polymorphism (n = 1 female, 8 males). Labels follow Table S3.

Statistic	plusminus	1000to50000	51000plus
aDNA			
Sites	620178	1076052	3606494
RADtags	5312	9329	31803
S	4650 (4520 - 4783)	8385 (8205 - 8572)	31344 (31000 - 31691)
θ_{W}	0.00218 (0.00212 - 0.00224)	0.00227 (0.00222 - 0.00232)	0.00253 (0.00250 - 0.00255)
π	0.00164 (0.00158 - 0.00169)	0.00172 (0.00167 - 0.00176)	0.00190 (0.00188 - 0.00193)
divergence	0.06114 (0.06050 - 0.06175)	0.06425 (0.06376 - 0.06474)	0.06672 (0.06646 - 0.06699)
π/divergence	0.027 (0.02582 - 0.02779)	0.027 (0.02602 - 0.02748)	0.029 (0.02813 - 0.02893)
TajD	-1.069 (-1.125 - -1.009)	-1.034 (-1.076 - -0.990)	-1.056 (-1.079 - -1.034)
Se/S	0.512 (0.498 - 0.526)	0.492 (0.481 - 0.503)	0.502 (0.496 - 0.507)
xDNA			
Sites	14076	24685	95369
RADtags	127	224	892
S	41 (29 - 54)	44 (31 - 57)	253 (225 - 285)
θ_{W}	0.00107 (0.00076 - 0.00141)	0.00066 (0.00046 - 0.00085)	0.00098 (0.00087 - 0.00110)
π	0.00094 (0.00066 - 0.00126)	0.00058 (0.00040 - 0.00076)	0.00085 (0.00074 - 0.00096)
divergence	0.05448 (0.05059 - 0.05896)	0.05228 (0.04931 - 0.05522)	0.05786 (0.05633 - 0.05961)
π/divergence	0.017 (0.01197 - 0.02328)	0.011 (0.00757 - 0.01474)	0.015 (0.01283 - 0.01663)
TajD	-0.626 (-1.131 - -0.087)	-0.565 (-1.116 - -0.004)	-0.691 (-0.895 - -0.484)
Se/S	0.585 (0.439 - 0.732)	0.591 (0.455 - 0.727)	0.565 (0.506 - 0.628)

Table S5: *M. maura* polymorphism (n = 2 females, 3 males). Labels follow Table S1.

Statistic	plusminus	1000to50000	51000plus
aDNA			
Sites	570287	1007276	3332818
RADtags	5198	9220	31199
S	2530 (2431 - 2643)	4469 (4345 - 4598)	16465 (16219 - 16718)
θ_{W}	0.00157 (0.00151 - 0.00164)	0.00157 (0.00152 - 0.00161)	0.00175 (0.00172 - 0.00177)
π	0.00156 (0.00149 - 0.00163)	0.00150 (0.00146 - 0.00155)	0.00169 (0.00166 - 0.00172)
divergence	0.06197 (0.06130 - 0.06267)	0.06494 (0.06440 - 0.06549)	0.06756 (0.06726 - 0.06784)
π/divergence	0.025 (0.02404 - 0.02635)	0.023 (0.02234 - 0.02385)	0.025 (0.02460 - 0.02546)
TajD	-0.032 (-0.111 - 0.043)	-0.214 (-0.270 - -0.157)	-0.161 (-0.192 - -0.127)
Se/S	0.397 (0.379 - 0.417)	0.442 (0.427 - 0.457)	0.423 (0.415 - 0.431)
xDNA			
Sites	9447	18642	65077
RADtags	101	198	760
S	15 (8 - 23)	30 (20 - 42)	101 (81 - 121)
θ_{W}	0.00076 (0.00041 - 0.00117)	0.00077 (0.00051 - 0.00108)	0.00074 (0.00060 - 0.00089)
π	0.00070 (0.00036 - 0.00108)	0.00076 (0.00049 - 0.00109)	0.00073 (0.00058 - 0.00087)
divergence	0.05305 (0.04828 - 0.05875)	0.05086 (0.04763 - 0.05426)	0.05987 (0.05803 - 0.06186)
π/divergence	0.013 (0.00677 - 0.02132)	0.015 (0.00971 - 0.02162)	0.012 (0.00974 - 0.01463)
TajD	-0.609 (-1.200 - 0.132)	-0.104 (-0.674 - 0.449)	-0.201 (-0.475 - 0.103)
Se/S	0.800 (0.600 - 1.000)	0.633 (0.467 - 0.800)	0.663 (0.574 - 0.752)

Table S6: *M. hecki* polymorphism (n = 4 females, 2 males). Labels follow Table S1.

Statistic	plusminus	1000to50000	51000plus
aDNA			
Sites	571933	999066	3375989
RADtags	5187	9182	31368
S	2957 (2851 - 3072)	5264 (5118 - 5395)	20390 (20110 - 20658)
θ_W	0.00171 (0.00165 - 0.00178)	0.00174 (0.00170 - 0.00179)	0.00200 (0.00197 - 0.00203)
π	0.00145 (0.00139 - 0.00151)	0.00149 (0.00144 - 0.00153)	0.00175 (0.00172 - 0.00178)
divergence	0.06186 (0.06119 - 0.06253)	0.06493 (0.06441 - 0.06545)	0.06735 (0.06705 - 0.06765)
π/divergence	0.023 (0.02240 - 0.02440)	0.023 (0.02221 - 0.02364)	0.026 (0.02554 - 0.02640)
TajD	-0.736 (-0.807 - -0.670)	-0.688 (-0.745 - -0.636)	-0.592 (-0.619 - -0.564)
Se/S	0.524 (0.506 - 0.542)	0.508 (0.495 - 0.521)	0.489 (0.482 - 0.496)
xDNA			
Sites	11500	21152	83301
RADtags	115	211	870
S	13 (7 - 21)	28 (18 - 38)	144 (121 - 168)
θ_W	0.00050 (0.00027 - 0.00080)	0.00058 (0.00037 - 0.00079)	0.00076 (0.00064 - 0.00088)
π	0.00048 (0.00023 - 0.00076)	0.00054 (0.00035 - 0.00076)	0.00071 (0.00060 - 0.00083)
divergence	0.05360 (0.04946 - 0.05778)	0.05168 (0.04858 - 0.05493)	0.05892 (0.05717 - 0.06061)
π/divergence	0.009 (0.00430 - 0.01429)	0.010 (0.00666 - 0.01511)	0.012 (0.01012 - 0.01412)
TajD	-0.244 (-1.069 - 0.668)	-0.410 (-0.953 - 0.243)	-0.404 (-0.640 - -0.130)
Se/S	0.615 (0.385 - 0.846)	0.643 (0.464 - 0.821)	0.653 (0.569 - 0.729)

Table S7: Parameter estmates from model fitting for *M. nemestrina* from Borneo ($n = 4$) polymorphism data. Models indicated with abbreviations and symbols that are defined in the Methods and discussed in detail in Evans et al. 2014. $\bar{x}$ indicates weighted average of parameter values.

model	θ_{01X}	θ_{10X}	γ_X	θ_{01A}	θ_{10A}	γ_A	λ	ρ_1	τ_1	$\ln L$	δAIC	wAIC
One Epoch												
∞	0.00195	0.00058	1.13186	0.00439	0.00114	1.44463	-	-	-	-3050429.949	357.59	0.000
Two Epochs												
full	0.00137	0.00049	0.93517	0.00253	0.00078	1.26642	2.313	2.359	0.652	-3050249.900	3.49	0.068
$\gamma_A = 0$	0.00125	0.00044	0.95318	0.00004	0.00004	0(fixed)	95.451	100.000	0.898	-3051716.354	2934.40	0.000
$\gamma_X = 0.75\gamma_A$	0.00106	0.00037	$0.75\gamma_A$	0.00254	0.00079	1.26558	0.75(fixed)	2.348	0.645	-3050250.156	0.00	0.386
$\gamma_X = 0$	0.00089	0.00080	0(fixed)	0.00253	0.00078	1.26648	2.405	2.360	0.653	-3050252.877	7.44	0.009
$\gamma_X = \lambda\gamma_A$	0.00115	0.00036	$\lambda\gamma_A$	0.00254	0.00078	1.26540	0.838	2.350	0.646	-3050250.108	1.90	0.149
$\lambda = 0.75$	0.00110	0.00036	1.02612	0.00254	0.00078	1.26545	0.75(fixed)	2.349	0.646	-3050250.139	1.96	0.145
$\theta_{01A} = \theta_{10A}$	0.00128	0.00046	0.91700	θ_{10A}	0.00144	0.08210	1.632	2.788	0.436	-3051539.332	2580.35	0.000
$\theta_{01X} = \lambda\theta_{10A}$	$\lambda\theta_{01A}$	0.00030	1.11559	0.00254	0.00078	1.26551	0.400	2.349	0.646	-3050250.332	2.35	0.119
$\theta_{01X} = \theta_{10X}$	θ_{10X}	0.00084	-0.10042	0.00253	0.00078	1.26641	2.356	2.359	0.652	-3050253.556	8.80	0.005
$\theta_{10X} = \lambda\theta_{10A}$	0.00099	$\lambda\theta_{10A}$	1.06700	0.00254	0.00078	1.26566	0.394	2.349	0.646	-3050250.336	2.36	0.119
$\bar{x}$	0.00108	0.00037	0.99594	0.00254	0.00078	1.26560	0.808	2.349	0.646			

Table S8: Parameter estimates from model fitting of *M. tonkeana* data ($n = 9$). Labels follow Table S7.

model	θ_{01X}	θ_{10X}	γ_X	θ_{01A}	θ_{10A}	γ_A	λ	ρ_1	τ_1	ln$L$	δAIC	wAIC
One Epoch												
∞	0.00139	0.00055	0.83380	0.00357	0.00105	1.28046	-	-	-	-4171971.390	4026.82	0.000
Two Epochs												
full	0.00072	0.00032	0.72472	0.00201	0.00077	1.02231	0.501	3.932	0.134	-4169956.509	3.06	0.075
$\gamma_A = 0$	0.00066	0.00029	0.73049	0.00117	0.00123	0(fixed)	0.524	4.565	0.140	-4171922.702	3933.45	0.000
$\gamma_X = 0.75\gamma_A$	0.00084	0.00035	$0.75\gamma_A$	0.00201	0.00077	1.02205	0.75(fixed)	3.925	0.135	-4169956.977	0.00	0.345
$\gamma_X = 0$	0.00052	0.00047	0(fixed)	0.00201	0.00077	1.02223	0.517	3.934	0.134	-4169963.181	14.41	0.000
$\gamma_X = \lambda\gamma_A$	0.00075	0.00036	$\lambda\gamma_A$	0.00201	0.00077	1.02267	0.630	3.928	0.135	-4169956.696	1.44	0.168
$\lambda = 0.75$	0.00081	0.00036	0.69784	0.00201	0.00077	1.02243	0.75(fixed)	3.925	0.135	-4169956.913	1.87	0.135
$\theta_{01A} = \theta_{10A}$	0.00066	0.00029	0.73020	θ_{10A}	0.00121	0.04892	0.510	4.544	0.136	-4171741.885	3571.81	0.000
$\theta_{01X} = \lambda\theta_{10A}$	$\lambda\theta_{01A}$	0.00027	0.84651	0.00202	0.00077	1.02178	0.339	3.940	0.133	-4169957.061	2.17	0.117
$\theta_{01X} = \theta_{10X}$	θ_{10X}	0.00048	-0.10748	0.00201	0.00077	1.02219	0.497	3.936	0.134	-4169965.323	18.69	0.000
$\theta_{10X} = \lambda\theta_{10A}$	0.00067	$\lambda\theta_{10A}$	0.69695	0.00202	0.00077	1.02264	0.390	3.937	0.133	-4169956.751	1.55	0.159
$\bar{x}$	0.00077	0.00033	0.73158	0.00201	0.00077	1.02229	0.606	3.930	0.134			

Table S9: Parameter estimates from model fitting of *M. maura* ($n = 6$) data. Labels follow Table S7.

model	θ_{01X}	θ_{10X}	γ_X	θ_{01A}	θ_{10A}	γ_A	λ	ρ_1	τ_1	$\ln L$	δAIC	wAIC
One Epoch												
∞	0.00104	0.00039	0.87006	0.00233	0.00089	1.05198	-	-	-	-3034844.889	0.00	0.477
Two Epochs												
full	0.00097	0.00038	0.83765	0.00203	0.00078	1.04255	14.460	1.157	5.000	-3034844.676	5.57	0.029
$\gamma_A = 0$	0.00029	0.00008	1.16941	0.00023	0.00025	0(fixed)	2.352	6.956	2.249	-3035631.009	1576.24	0.000
$\gamma_X = 0.75\gamma_A$	0.00100	0.00041	$0.75\gamma_A$	0.00234	0.00089	1.05618	0.75(fixed)	0.010	0.003	-3034844.769	1.76	0.198
$\gamma_X = 0$	0.00066	0.00060	0(fixed)	0.00201	0.00078	1.04193	14.455	1.168	5.000	-3034847.515	9.25	0.005
$\gamma_X = \lambda\gamma_A$	0.00104	0.00039	$\lambda\gamma_A$	0.00234	0.00089	1.05588	0.832	0.010	0.004	-3034844.741	3.70	0.075
$\lambda = 0.75$	0.00102	0.00039	0.86792	0.00229	0.00088	1.04768	0.75(fixed)	1.022	1.223	-3034844.839	3.90	0.068
$\theta_{01A} = \theta_{10A}$	0.00090	0.00037	0.79876	θ_{10A}	0.00118	0.08340	3.370	1.416	1.095	-3035506.559	1327.34	0.000
$\theta_{01X} = \lambda\theta_{10A}$	$\lambda\theta_{01A}$	0.00039	0.87835	0.00234	0.00089	1.05552	0.446	0.010	0.003	-3034844.767	3.76	0.073
$\theta_{01X} = \theta_{10X}$	θ_{10X}	0.00063	-0.10469	0.00202	0.00078	1.04236	14.816	1.161	5.000	-3034848.278	10.78	0.002
$\theta_{10X} = \lambda\theta_{10A}$	0.00104	$\lambda\theta_{10A}$	0.87110	0.00234	0.00089	1.05555	0.443	0.010	0.003	-3034844.767	3.76	0.073
$\bar{x}$	0.00103	0.00040	0.84863	0.00232	0.00089	1.05298	0.851	0.116	0.266			

Table S10: Parameter estimates from model fitting of *M. hecki* data ($n = 6$). Labels follow Table S7.

model	θ_{01X}	θ_{10X}	γ_X	θ_{01A}	θ_{10A}	γ_A	λ	ρ_1	τ_1	ln$L$	δAIC	wAIC
One Epoch												
∞	0.00098	0.00045	0.68700	0.00294	0.00089	1.28375	-	-	-	-3073983.524	744.36	0.000
Two Epochs												
full	0.00067	0.00034	0.57742	0.00213	0.00078	1.09147	0.502	2.526	0.132	-3073609.850	3.01	0.063
$\gamma_A = 0$	0.00064	0.00032	0.57000	0.00121	0.00131	0(fixed)	0.614	2.890	0.155	-3074877.126	2535.57	0.000
$\gamma_X = 0.75\gamma_A$	0.00081	0.00032	$0.75\gamma_A$	0.00213	0.00078	1.09019	0.75(fixed)	2.517	0.134	-3073610.343	0.00	0.287
$\gamma_X = 0$	0.00051	0.00046	0(fixed)	0.00213	0.00078	1.09144	0.502	2.528	0.132	-3073612.043	5.40	0.019
$\gamma_X = \lambda\gamma_A$	0.00068	0.00035	$\lambda\gamma_A$	0.00213	0.00078	1.09148	0.521	2.525	0.132	-3073609.852	1.02	0.172
$\lambda = 0.75$	0.00073	0.00037	0.57960	0.00213	0.00078	1.09133	0.75(fixed)	2.517	0.134	-3073609.974	1.26	0.153
$\theta_{01A} = \theta_{10A}$	0.00064	0.00032	0.56993	θ_{10A}	0.00127	0.08219	0.595	2.895	0.150	-3074690.970	2163.25	0.000
$\theta_{01X} = \lambda\theta_{10A}$	$\lambda\theta_{01A}$	0.00029	0.64843	0.00214	0.00078	1.09148	0.287	2.542	0.129	-3073610.134	1.58	0.130
$\theta_{01X} = \theta_{10X}$	θ_{10X}	0.00048	-0.10659	0.00213	0.00078	1.09144	0.495	2.528	0.132	-3073612.931	7.18	0.008
$\theta_{10X} = \lambda\theta_{10A}$	0.00064	$\lambda\theta_{10A}$	0.56548	0.00213	0.00078	1.09166	0.422	2.531	0.131	-3073609.877	1.07	0.168
$\bar{x}$	0.00070	0.00034	0.63586	0.00213	0.00078	1.09112	0.573	2.525	0.133			

Table S11: Weighted average of parameter estimates over all models for *M. nemestrina* from Borneo, *M. hecki*, and *M. tonkeana*. Model averages are not reported for *M. maura* because the equilibrium model was not rejected and the λ parameter therefore could not be estimated (Supplementary Table S9). As described in the Supplement, θ_{01X} is the polymorphism parameter for sites on the X where the derived mutation is an A or T, θ_{10X} is the polymorphism parameter for sites on the X where the derived mutation is an G or C, γ_X is the selection parameter for GC content on the X. θ_{01A}, θ_{10A}, and γ_A are the corresponding parameters for the autosomes. λ, ρ_1, and τ_1 refer respectively to the X/A polymorphism ratio, the ratio of the current to ancestral population size, and number of generations before the present that the population size changed

Species	θ_{01X}	θ_{10X}	γ_X	θ_{01A}	θ_{10A}	γ_A	λ	ρ_1	τ_1
M. nemestrina Borneo	0.00108	0.00037	0.99594	0.00254	0.00078	1.26560	0.808	2.349	0.646
M. hecki	0.00070	0.00034	0.63586	0.00213	0.00078	1.09112	0.573	2.525	0.133
M. tonkeana	0.00077	0.00033	0.73158	0.00201	0.00077	1.02229	0.606	3.930	0.134

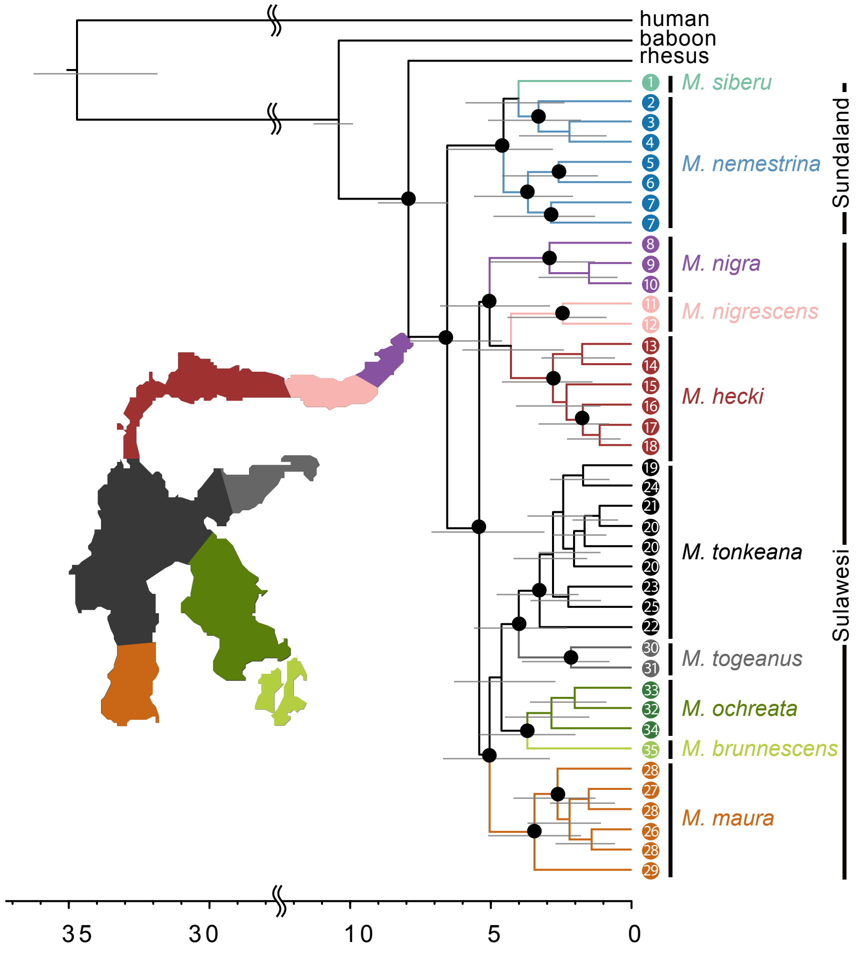

Figure S1: Chronogram estimated from RADseq data from the X chromosome. Labels follow Fig 2.

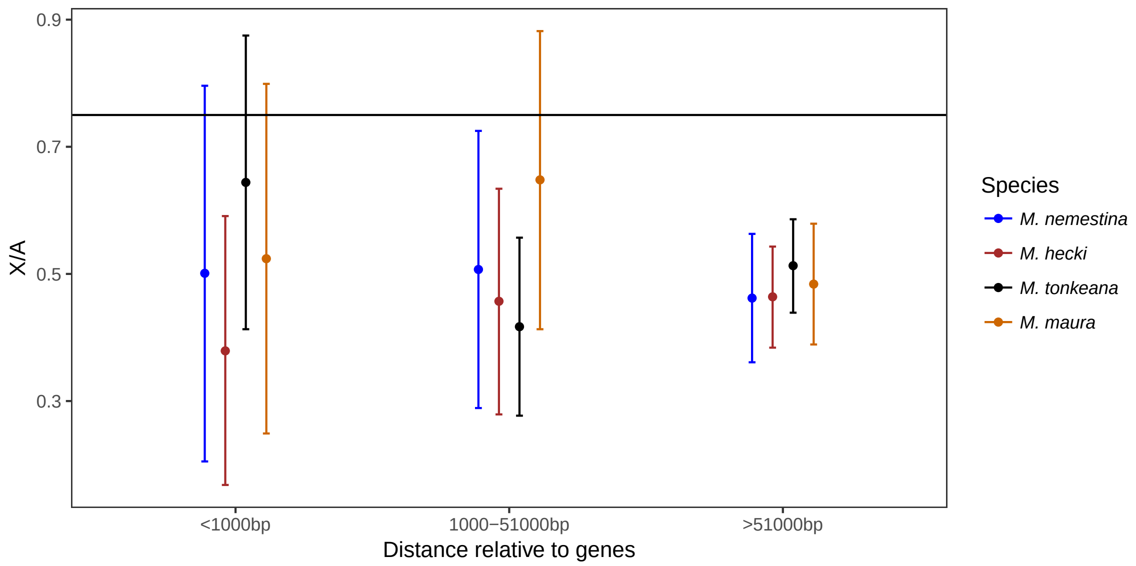

Figure S2: X/A polymorphism ratios (X/A) based on RADseq data for four species. Ratios were calculated in three genomic categories: (1) exonic and intronic sequences and flanking regions less than 1000 bp from genes (<1000bp), (2) nongenic regions that are between 1,000 and 51,0000 bp from genes (1000–51000bp), and (3) nongenic regions that are greater than 51,000 bp from genes (>51000bp). Bars indicate 95% confidence intervals and accommodate uncertainty in polymorphism and divergence. A horizontal line indicates the 0.75 expectation.

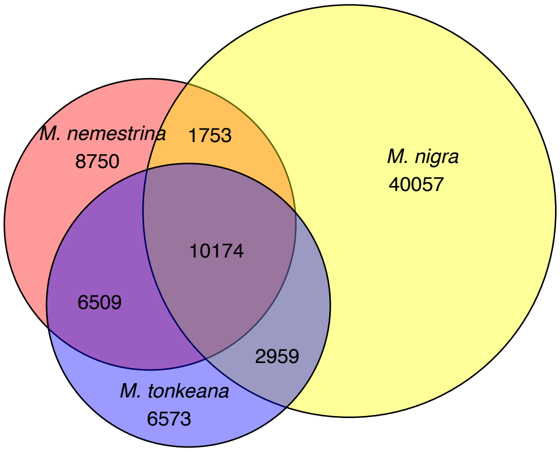

Figure S3: A Euler diagram of the number of sites in the non-pseudoautosomal region of the X chromosome for which a heterozygous genotype was inferred for one or more of the three individuals used in the analysis of gene flow across Wallace's Line. Numbers in each region of the chart refer to the number of shared or unshared heterozygous genotypes, and is based on ~57.5 million genotyped sites in each individual after discarding repetitive regions. Most of the (pseudo-) heterozygous genotypes in each male were shared with the other, whereas most of the heterozygous genotypes in the female were not shared with either male. This analysis is consistent with the proposal that most of the heterozygous genotypes in the female are real.
